# A Question of Choice

**DOI:** 10.1186/1742-4755-8-20

**Published:** 2011-06-22

**Authors:** Gene Grabiner

**Affiliations:** 1 SUNY Distinguished Service Professor, Retired 359 Parkside Avenue Buffalo, New York 14214, USA

## Abstract

Women's reproductive rights, reproductive health, and constitutional privacy rights in the United States are addressed in light of the contemporary onslaught of the Christian Right. The misuse of State power by fundamentalist social forces in America is critiqued. The article also briefly reviews the question of State control over women's bodies.

## Introduction

Freedom and self-determination are under attack by the Christian Right in the United States today. The defense of a woman's right to end an unwanted pregnancy must be part of everyone's larger struggle to protect our civil, political and human rights. Central to the question of a woman's right to choose to have children or not is the tension between our American Enlightenment-democratic traditions of the rights of the individual opposed to intrusive State power. The Catholic Church hierarchy has employed lobbying, picketing of reproductive health clinics and Papal Encyclicals to oppose abortion rights. Fundamentalist Christians, comprising another wing of the religious right, use intimidation tactics, violence, and attempts to induce State domination of reproductive rights. The anti-choice ideology and practice of both the Catholic Church hierarchy and fundamentalist Christians constitute the Christian Right, and highlight the anti-democratic character of their movement.

## The State and Reproductive Rights

Loss of choice cuts two ways. By giving the government the power to prevent, for example, the choice of abortion, the government also acquires the power to require abortion or more extreme measures if its political "mood" were to shift directions. If today, choice is made illegal and predominantly low income, working-class, and women of color are forced by the State to bear children, the State may, at the same time, or later, also deny certain women, perhaps the very same women, the right to bear children at all. This has already happened in modern history

A memorandum to Adolf Eichmann from Nazi-occupied Poznan, Poland noted that "...all the Jewish women, from whom one could still expect children, should be sterilized so that the Jewish problem may actually be solved completely with this generation." [1] And, from the Nazi-occupied Netherlands we read that:

*For the remaining Jews and Jewesses the aspired goal is voluntary sterilization which is to be carried out in Amsterdam. In case of refusal, forced sterilization should follow in Camp Hertogenbosch*[2].

The anti-choice view that woman's destiny is to bear children, and to be legally forced to bear children, echoes earlier, barbaric visions of woman's place in society - to remain "barefoot and pregnant" or to be solely concerned with "*kinder *(children), *kuüche *(kitchen) and *kirche *(church)," as the Nazis put it. It may also be remarked that while the Nazis sterilized Jewish women, "Aryan" women were simultaneously urged to reproduce and were rhetorically and ideologically ennobled for doing so.

The United States has a deplorable history of sterilization abuse of women of color and people with mental illness or disabilities. For example, in the early part of the 20th century, Native American and African American women were sterilized against their will in many states. This typically occurred without their knowledge and consent while they were in a hospital for other surgeries, such as C-sections[3]. Poor white women were similarly victimized in our southern states.

Recall those American jurists who required certain women to be sterilized because they were declared to be "feeble-minded." In 1927, Chief Justice of the Supreme Court Oliver Wendell Holmes, writing for the majority in support of involuntary sterilization in *Buck v. Bell*, observed that "[t]hree generations of imbeciles are enough." [4] It is estimated that, in the twentieth century, more than 65,000 individuals were sterilized in 33 states under state compulsory sterilization programs in the United States[4].

More recently, other jurists have required certain women to be sterilized in order to receive public assistance. In the 1960s and 1970s, at least ten states proposed legislation, (not passed), to force women on welfare to use birth control[5]. We should also never forget the United States' government sterilization campaign in Puerto Rico. By 1965, it was estimated that 34% of all Puerto Rican women between the ages of 20-49 years had been sterilized[5]. In 1973, the Southern Poverty Law Center released the fact that two African American girls were sterilized in Alabama without their knowledge or consent. Bureau of Indian Affairs hospitals were believed to be "particularly egregious in their abuse of sterilization." [5]

Laws were passed to prevent such reproductive abuses. Yet, in the 1990s, after the Food and Drug Administration approved the contraceptive implant, Norplant, judges and legislators tried to use it to control women's reproduction. They tried to mandate the use of Norplant by women on welfare with several children in order to prevent them from conceiving for five years. Efforts "to coerce women to use Norplant represent a reversion to an era of overt racism and eugenics." [6]

Whether the State forces a woman to give birth or forces her not to, there is in either case, no right to individual choice or privacy.

## Choice

The attack on abortion as a privacy right in the United States was launched immediately after the Supreme Court's landmark decision in *Roe v. Wade*

When the Supreme Court legalized abortion in 1973, the anti-abortion forces, led initially by the Catholic Church hierarchy, began a serious mobilization using a variety of political tactics including pastoral plans, political lobbying, campaigning, public relations, papal encyclicals, and picketing abortion clinics. The Church hierarchy does not truly represent the views of U.S. Catholics on this issue or the practice of Catholic women, who have abortions at a rate slightly higher than the national average for all women[7].

Catholic Church social teaching also refers to human embryos as "children in the womb." Despite these early and continuing attacks on women's abortion rights, and the redefinition of the embryo as child, the Fourteenth Amendment to the American Constitution holds that "All persons *born *[my emphasis] or naturalized in the United States and subject to the jurisdiction thereof are citizens of the United States and of the state wherein they reside." The wisdom of the Fourteenth Amendment acknowledges personhood as a social condition conferred at birth with consciousness and more fully realized through social development. Certain religious denominations also proclaim personhood at birth. Many major religious groups support a woman's reproductive rights. But, fundamentalist Christian groups, such as Operation Rescue, along with the Catholic Church hierarchy, claim that "ensoulment" occurs at conception and that the fetus is a person or unborn child. They influence legislators to pass anti-abortion laws in many states in the United States in a concerted effort to make their view the law of the land, conferring both religious intolerance and hostility to Fourteenth, Fourth, and First Amendment freedoms. By 2003, 23 states had introduced legislation redefining the fetus as a person[8]. Currently, 38 states have fetal homicide laws. But of these, 22 declare the fetus to be a "person," "homo sapien," "human," or "human in utero." [9]

What are the political implications of many states' legislated redefinitions of the fetus as a "human person?" In the Fourteenth Amendment, the primary citizen relation is between the individual and the national government. State jurisdiction over the individual is secondary or derivative. But, if a renovated states rights theory of government becomes law, despite persisting legislative defenses of democracy (such as the Fourteenth Amendment's subduing of states' rights theory), then, the direct relation between Americans and their Constitution would be broken.

## The Economics of Anti-Choice

The extent of unemployment in a society helps determine the average wage. If there are fewer jobs than workers, the average wage will fall. And if there are more jobs than workers, the average wage will rise. Before the recent Great Recession (which for many is still a continuing Depression), theorists of a coming American labor scarcity had expected wage increases among the ranks of the poor, minorities, and the low-skilled. Now, however, coupled with persistent unemployment, if American birthrates rise, (perhaps as a function of outlawing choice), the predicted job shortage will not occur and American wages will decline even more.

The Guttmacher Institute in November 2010 [10] reported that because of economic hardship, nearly half of low- and middle-income women wanted to delay pregnancy or limit the number of children they have, but that many had to skimp on their contraceptive use--or forgo it entirely-- to save money. Publicly funded family planning providers were having difficulty meeting the need for subsidized contraceptive care. Conservative federal legislators have already mounted a campaign to eliminate Title X funding for family planning, mammograms and other needed reproductive health care for low income and young women across the nation. Although, as of this writing, they failed to cut Title X from the budget, this progressive "victory" came with an unacceptable compromise of additional budget cuts from other desperately needed social welfare areas. The assault has been temporarily stopped at the federal level. But it is more than likely that conservative states will take up the call again in their own legislatures.

Currently, America's capital has been victimized by this fundamentalist, (and Tea Party), attack. In order to achieve a "compromise" $38.5 billion federal budget cut, the Obama administration agreed to deny Washington, DC women the right to use "congressionally appropriated funds, whether federally or locally generated," to secure abortions when necessary. The DC mayor, Vincent Gray, was arrested along with 36 others while protesting this abortion funding ban[11].

Apart from the increased Hispanic/Latino teen-age birthrate (2005 data) [12], the greatest overall increase in the American birthrate, consequent to outlawing choice, is likely to occur among poor and working people, many of whom are already unemployed/underemployed. So, anti-choice would further victimize already hard-hit families while potentially expanding the pool of cheap labor through an enormous increase in births.

While overall, the American birthrate has largely declined in the past few years; our crumbling infrastructure can barely handle even our existing numbers. If the interlocking and wealthy fundamentalist movement and the Catholic Church have their way, who will provide jobs, food, housing, education, transportation and medical care for the millions more that would further strain an already failing infrastructure? If the right-wing billionaire Koch Brothers and New Jersey, Ohio, and Wisconsin governors Christie, Kasich and Walker are bellwethers, there is no longer any social requirement to provide jobs, food, housing, education, transportation and medical care etc. in America. The social contract has been broken through manipulation of states' rights legislation.

## Health Related Issues

When abortion was illegal in the United States, approximately 1,000,000 abortions were performed annually[7]. Most were performed in unsafe and/or unsanitary conditions and without medical care-- often resulting in injury or infection, sometimes in death. In just the first decade since *Roe v. Wade *alone, legal abortions averted at least 1500 or more pregnancy - related deaths and tens of thousands of life-threatening complications[13].

## More War on Women, Anti-Choice Contributions to Illegal Abortion

Choice means either having or not having a baby. For leaders of the Christian Right, of course, there is no choice and abortion must be criminalized. Indeed, recently proposed South Dakota legislation, (February, 2011), would not only criminalize abortion, it would declare it to be a justifiable homicide if one were to kill an abortion provider. "House Bill 1171 would expand the legal definition of justifiable homicide in the state. Critics said the measure legalizes the killing of abortion providers by saying a homicide is permissible, if committed by a person "while resisting an attempt to harm" an unborn fetus." [14]

Last year, the Utah legislature passed a bill criminalizing women who have induced miscarriages or miscarriages that occur due to "reckless" behavior. "The bill passed by legislators amends Utah's criminal statute to allow the state to charge a woman with criminal homicide for inducing a miscarriage or obtaining an illegal abortion[15]. Due to mass opposition, the governor of Utah, a Republican, did not sign the bill. For now, it has returned to the legislature, from whence it may yet reappear.

The defeat of Georgia Senate Bill 529 was a bright spot among some 600 bills reducing abortion access introduced in state legislatures in 2010. The bill's authors falsely assumed that most women seeking abortion are coerced to do so; that they couldn't be trusted to make decisions for themselves, and that they could not think with their own minds about choices they would make about their own bodies. Additionally, concomitant with a campaign in the African American community, mentioned in the quote below, the bill would have criminalized physician- performed abortions "that had allegedly been 'coerced' due to the race or sex of the fetus." Georgia SB529 was beaten by SisterSong and their allies.

Women of color and our allies won a significant policy victory in Georgia in 2010 when we successfully challenged a dangerous publicity campaign launched with billboard-splashed allegations that claimed "Black Children are an Endangered Species." We defeated state legislation attempting to expand abortion restrictions by linking race, gender and abortion...Georgia Senate Bill 529 - failed due to the leadership of women of color working together using the reproductive justice framework.

...our intersectional approach effectively contested this new front in the abortion wars that would have wielded race and gender as a weapon to undermine abortion rights. By working strategically with pro- choice allies in our state and across the country, we brought our collective resources together to deliver one of the few national success stories in the 2010 legislative season. Women of color groups played a unique and essential role as a trusted voice that galvanized leaders of color and key civil and human rights organizations to take action on abortion rights issues, sometimes for the first time. We proved that leaders and institutions that needed to be activated in the African American community could only be moved by black women working together.

*Our vision for the future is that national Civil Rights organizations officially and publicly join the Reproductive Justice movement in defending the human rights of women of color*[16].

While anti-choice groups claim to oppose both legal and illegal abortions, they are not ignorant of the realities of American life. For those women who could not afford to leave this country to have a legal and medically safe abortion, outlawing abortion would simply revive the unsafe and possibly fatal practices of the past. Since recriminalizing abortion and/or overturning *Roe v*. *Wade *would certainly not prevent abortions, an environment would be created in which back alley abortions would again flourish in the United States. Nationwide, the health effects on poor women and women of color, who live in states that already have severely restricted access to abortion, would be even more devastating[17]. RU-486 and emergency contraception are also in the anti-abortionists' sights.

While RU-486, the so-called "abortion pill" has only recently been legalized in the United States, it has been available for some time in France, a more heavily and titular Catholic country. Entirely consistent with First and Fourth Amendment freedom from State religion, religious freedom, and privacy rights, or civil and human rights, RU-486 makes abortion a fully private matter consistent with the legal theory undergirding *Roe v*. *Wade*. RU486 is not the morning after pill which is emergency contraception. Emergency contraception does not cause an abortion. Yet, the religious right persists in inaccurately attacking emergency contraception as capable of causing an abortion. Emergency contraception is a form of contraception that can be used immediately after unprotected sexual intercourse, but before pregnancy is established. Pharmacists in some states in the US refuse to stock emergency contraception either because they mistakenly believe it can cause an abortion or have been intimidated by the growing power of the religious right.

## What does it mean to be Pro-Life? Who is Pro-Life?

Who may properly claim the title "pro-life"? Shakespeare says that "you take my life when you take the means whereby I live." And so, life is shaped by the social conditions into which the fetus comes as a newborn. With but few exceptions, it is precisely at birth that the Christian Right and their supporters abandon the infant. Such children may not literally be abandoned by the families into which they are born. But, if they are poor families forced by anti-abortion laws to become multi-child families what may be expected of the quality of life for those children? There is no evidence that the Christian Right stands ready to provide enhanced quality of life for all of them via adoption or by supplementing their parents' meager resources.

This is also a question of simple numbers. If the anti-choice forces triumph with a new "baby boom," Catholic Charity coffers and Christian Right dollars would not be up to the task of providing adequate support for many, if not most, of these children; children who might then never be able to fulfill their potential due to inadequate economic, social and educational conditions.

The overwhelming majority of Americans are still pro-choice and, therefore, truly pro-life. So, it may be said that the Christian Right's commitment to life begins with the fetus and ends with birth. And while they oppose infanticide re-defined by them as abortion, through silence or avoidance of the issues they fail to address the larger questions of infant and child health and well-being. Such issues are directly related to broad social access to health care, quality affordable child care, nutrition, housing, education, etc. In fact, these are among the programs that conservative politicians and those on the Christian Right would also like to see slashed in order to balance the U.S budget.

It may be said that with their rabid focus on control of women, anti-choice groups reduce humanity to less than the animal level; or at least to less than the level of the higher animals, those which exhibit social learning and care for their offspring until they may fend for themselves. At the level of the lowest animal life, instinct dominates. Humans, however, do not have instincts. They require social development. Hence, to demand for us the yardstick of nature alone is, therefore, a process of dehumanization.

Since anti-choice groups' only commitment lies with a strictly biological/natural/animal process -- bringing the fetus to full term -- they effectively reject complete caring for children which, of course, includes social development. Yet, a newborn is always still a "candidate for hominization"-- becoming fully human. Socialization is the process by which we become fully developed human beings. Brain growth continues after birth; and synapse development in the brain continues after birth and requires social stimulation to occur. Without such stimulation, infants often die or exhibit "failure to thrive" syndrome. That stimulation, among other things, may include a nurturing environment, quality child care, health care and education, etc.

## Hypocrisy, Anti-Choice, and Violence

There may be a few in the anti-choice movement who are consistent champions of life and oppose the death penalty and war, and support both the private and publicly sponsored and funded protection and adoption of unwanted children. The majority of anti-choicers, however, seem to show false compassion; and some have used terroristic violence in their cause.

Specifically, a number of those in the fundamentalist anti-choice movement have supported and/or been directly involved in: hostile "in your face" picketing or blockading of abortion clinics, clinic invasions, vandalism, trespassing, arson and bombings, saturation of clinics with vomit-smelling butyric acid, and assault and battery of clinic staff and providers. Some of these champions of "life" have even resorted to murder[18].

From October 1993 through May 2009, four physicians who performed abortions were killed by members of the Christian Right.

In the first such murder, on Thursday, March 11, 1993, obstetrician/gynecologist Dr. David Gunn was gunned down at point-blank range in front of his clinic by one of a group of assembled "pro-life" demonstrators[19].

Within one year and a half, Dr. John Britton was murdered along with his body guard by anti-abortion activist, Paul Jennings Hill. Hill, founder of Defensive Action, a group that advocated violence against abortion providers was an excommunicated Presbyterian minister affiliated with the Christian terrorist Army of God organization[20-23].

On October 23, 1998, Dr. Barnett Slepian, a Buffalo, New York obstetrician/gynecologist, had just returned from his synagogue after attending a memorial service for his father. He was preparing dinner for his family when he was shot through the window of his home with a high-powered rifle fired by James C. Kopp, an acolyte of the "Lambs of Christ." "The bullet shattered" Dr. Slepian's "spine and tore his aorta, barely missing his son's head as it exited. He died two hours later." Kopp was also a suspect in the shooting of four other doctors in Canada and in Rochester, New York. In those instances, none of the victims died[24-26].

On Sunday, May 31, 2009, Dr. George Tiller was shot to death while acting as an usher at the Reformation Lutheran Church in Wichita, Kansas. His clinic had previously been bombed; and he had previously been shot in both arms by an abortion opponent[27].

In addition to these physician murders, anti-choice terrorists have killed abortion clinic receptionists and security guards.

For an up-to-date overview of violence and disruption against abortion clinics and providers, in the United States and Canada, it is recommended that the reader consult the National Abortion Federation statistics[28].

In addition to committing violence against abortion clinics and providers, Christian fundamentalists tend to support the death penalty, war, militarism, and empire-building. They do this all while championing biological obligation, i.e., legally forcing pregnant women to bear children. Yet, at the same time, they reject social obligation. That is, they do not actively call for publicly supported quality health care, child care, education, housing and jobs for the poor, lower and middle income people and women of color - those who would be most affected by a reversal of *Roe v*. *Wade*. Indeed, they ought to be fully consistent and realistic about the broad social consequences of unplanned childbirth.

There is another sense in which the religious right is hypocritical. No doubt many of them hew to classical American individualism as a matter of principle and are opposed to the intrusion of the State into their own private lives. Yet, out of a basic opposition to religious freedom, they assert that the United States is a "Christian country." And, in opposition to the American individualism of reproductive choice, they would impose intrusive State power on others.

This willingness to intrude has also occurred in health care and outside of the role of the State. For financial reasons, some Catholic hospitals or health systems have been sold to secular hospitals and health systems in different states. These sales have included codicils requiring that the new owners "continue to follow Catholic health restrictions." Consequently, these hospitals and health systems refuse to provide abortions and even referrals to physicians who perform them for women who want them. Many of these hospitals have been prevented from using standard medical treatments for women, even in emergency situations, such as treatment of ectopic pregnancy with a drug that is also used to induce abortion. Due to persisting Catholic Church domination of these hospitals, many also fail to routinely offer emergency contraception to women who have been raped[29]. Clearly, it's not only a question of abortion. The religious right is having a broadly negative effect on women's human right to health and health care.

What of anti-choice men, those who are invariably in the lead in these Christian anti-abortion organizations and who don't believe that a woman should have reproductive rights? They are an interesting group, primarily because, on the one hand, they oppose a woman's right to choose and oppose abortion as one possible expression of that choice, yet, on the other hand, that is a choice they will never have to face. They are ready to prescribe, but they cannot partake.

## Racism and Anti-Choice

If choice were outlawed, among those most harmed would be working class, low income, and poor white and minority women; those who can least afford unwanted children, and who would be most victimized by illegal abortions. Although some people of color are apparent in videotapes of actions at abortion clinics, Operation Rescue has few minority group members. And there is still a racist dimension to the anti-choice movement.

Since birth rates for racial/ethnic minorities in America exceed those for majority whites/Anglos, (with the Hispanic/Latino birth rate now exceeding the black and Native American rates), some argue that increasing the white birth rate is an unspoken goal of the predominantly white anti-choice movement. Many demographers predict that America will be a mostly brown nation by mid-21st century. Anecdotally, abortion clinic escorts and guards have reported that anti-abortion protestors appear less passionate in their appeals to black women than to white women at these sites. Yet, only in the past two years in what appears as a pitch to the black community, some right wing groups have posted giant billboards in the state of Georgia and elsewhere equating abortion with genocide of African American babies[16].

Against the backdrop of the increasing "feminization of poverty," the burdensome prescription of more births falls heaviest on poor and working class Hispanic/Latino and African American women; those whose men are most disproportionately incarcerated, or under some sort of police control, or unemployed in America.

For example, as of 2003, one in nine black men aged 25-29 was in prison or in jail in the United States[30]. And,

[i]n the late 1990s, nearly one in three African American men aged 20-29 were under criminal justice supervision, while more than two out of five had been incarcerated - substantially more than had been incarcerated a decade earlier and orders of magnitudes higher than that for the general population. Today, 1 in 15 African American children and 1 in 42 Latino children have a parent in prison, compared to 1 in 111 white children. In some areas, a large majority of African American men - 55 percent in Chicago, for example - are labeled felons for life, and, as a result, may be prevented from voting and accessing public housing, student loans and other public assistance[31].

Increased imprisonment of African American men is largely a result of America's so-called War on Drugs policies. Harsh "3-strikes" rules, meaning commission of three crimes, in some states, have condemned men to imprisonment for life, even for non-violent crimes.

Additionally, joblessness for 16-to-24-year-old black men has reached Great Depression proportions -- 34.5 percent[32]. Young black women have an unemployment rate of 26.5 percent[33]. While not quite as dramatic, Hispanic unemployment statistics are equally powerful. As of May, 2011, of all Hispanic women ages 20 and over, 11.1 percent are unemployed. For Hispanic men, the number stands at 10.1 percent[34].

The emotional stress of having a father or husband in prison added to the economic stress of being unemployed with children can only be exponentially magnified, if women are denied the right to decisions to have or to not have additional children.

## Anti-Choice and Ecocide

Unplanned population growth in the context of unregulated production for the capitalist market places increasing demands on environmental carrying capacity. This is nowhere more glaring than in the developing world. Wary of this problem, amidst a history of global, colonial and indigenous resource looting and mismanagement, some low income countries have instituted national family planning programs. Making choice illegal in America, however, would increase population and population density; quite likely even further draining third-world and domestic resources and intensifying various world ecological crises. Raw materials and finished goods are imported to the United States, in many cases disallowing production of basic necessities in source countries.

## Conclusion: Reproductive Freedom, Abortion and Family Planning

In an age of AIDS crisis, the more than 50% of all American pregnancies that are unintended reflect lack of access to more effective contraceptive methods. Abstinence- only programs have been demonstrated not to work, yet millions of taxpayer dollars have been given over to mostly religious organizations to implement such one-sided approaches to sexuality education[35,36]. Many women live in circumstances that prevent them from controlling the context in which they have sex and if they use contraceptives or not. Unintended pregnancy is also the result of sexual coercion of young women, forced sex and rape, or situations where contraceptives fail--none are 100% effective.

In the United States, unlike in other Western countries, the state public health system is not conducive to promoting modern, more effective methods of contraception. Nor is there broad-based, accessible mass education about family planning and sexuality education for young people. Contraceptive information, including basic, clear and objective messages regularly presented in the mass media, social agencies, schools, and workplaces, is just not available. Many health insurance companies do not cover contraception, which can be costly. For example, the IUD Mirena and the medical visit for insertion may cost as much as $700.00, making it virtually out of reach for low income women. These circumstances may, in part, help to explain America's high incidence of unplanned and unwanted births.

Democracy is fragile. Today it is under attack by fascist ideologues and varieties of Christian fundamentalists. This general hostility to democracy is also a hallmark of the Christian Right.

And so, in defending our sisters' right to choice, we defend ourselves, and we defend democracy for all.

## Competing interests

The author declares that he has no competing interests.
